# Opportunities and challenges in delivering maternal and child nutrition services through public primary health care facilities in urban Bangladesh: a qualitative inquiry

**DOI:** 10.1186/s12913-023-10094-6

**Published:** 2023-10-27

**Authors:** A M Rumayan Hasan, Mohammad Abdus Selim, Faugia Islam Anne, Jessica Escobar-DeMarco, Santhia Ireen, Kristen Kappos, Deborah Ash, Sabrina Rasheed

**Affiliations:** 1 Health Systems and Population Studies Division (HSPSD), International Centre for Diarrhoeal Disease Research, 68 Shaheed Tajuddin Ahmed Sarani, 1212 Mohakhali, Dhaka Bangladesh; 2 Department of Public Health Sciences, The University of North Carolina Charlotte, Charlotte, NC USA; 3 FHI Solutions / Family Health International 360, Washington, DC USA

**Keywords:** Primary health care, Nutrition services, Barriers, Facilitators, Urban, Bangladesh

## Abstract

**Background:**

Public primary health facilities are an important source of nutrition services for the urban areas in Bangladesh. We aimed to understand the challenges and facilitators of delivering maternal and child nutrition services through public sector from the perspectives of the users and service providers.

**Method:**

The study was conducted in selected public primary health care facilities and their catchment area in Dhaka city from April-July 2019. We carried out 15 free listing exercises and 43 semi-structured interviews (SSI) with pregnant women and mothers of 0–24 months old children; 6 key informant interviews (KII) with facility managers and healthcare providers; and observed service delivery in 8 health facilities.

**Results:**

Findings reveal that public primary health facilities address some economic and cultural barriers to access such as cost and provision of female service providers for maternal and child health services but challenges such as distance, waiting time, and cleanliness remained. In terms of service provision, there were gaps in provision of anthropometric measurement and counseling, and healthcare providers had inadequate training and therefore, knowledge of nutrition. The low priority given to nutrition services during program design hampered the delivery of nutrition services provided through urban public sector health facilities.

**Conclusions:**

There were important gaps in terms of service provision and capacity of healthcare providers, and therefore, the quality of nutrition service provided through public primary health care facilities. To maximize the coverage of quality nutrition services in the urban areas, it is important to think through the design of nutrition service delivery and allocate adequate resources to fill the material and capacity gaps.

**Supplementary Information:**

The online version contains supplementary material available at 10.1186/s12913-023-10094-6.

## Background

The urban population has increased in Bangladesh from 6 million in 1974 to 52 million in 2022 [1, 2] and about 31.5% of the total population of the country now live in urban areas [1]. Among the urban residents, 1.8 million [1] live in informal settlements which are characterized by overcrowding, limited access to safe drinking water, hygiene and sanitation [3, 4]. High levels of malnutrition and low utilization of maternal and childcare services among the urban poor indicates the existing health inequity [5, 6]. In terms of malnutrition, although among general urban population 26.3% of children < 5 years of age are stunted, among urban poor the level of stunting was 40% [7]. Similarly the urban poor had significantly lower rates of Antenatal Care (ANC) utilization (39.8%vs 53.1%), facility delivery (53.7% vs. 77.3%) [8] and immunization (88.5%% Vs 75%) [3] compared to the general urban residents. Despite the need for primary healthcare services the urban poor tend to use private healthcare facilities more compared to public healthcare facilities for ANC services (53% vs. 23%) [8]. As nutrition services are provided through the public primary healthcare facilities, it is important to understand the state of nutrition services provided through them so that they can serve the urban residents especially the poor better.

To address the high levels of malnutrition among the Bangladeshi population, nutrition services were implemented from 1996 to 2011 (Bangladesh Integrated Nutrition Program and National Nutrition Program) as standalone programs by the Bangladesh Government [9]. These programs We criticised for lack of coordination and linkages with existing health services, expensive administration cost, and minimal effect on improving maternal and child nutrition indicators [10]. From 2011, nutrition services were mainstreamed within health services under the National Nutrition Services (NNS) Operational Plan of the Health, Population, and Nutrition Sector Development Programme (HPNSDP) [11]. Despite the mainstreaming, a study conducted in 2017 demonstrated that the quality of nutrition services provided by the public sector in rural areas was inadequate [12]. In urban areas of Bangladesh, the governance of the health service provision in the public sector is complex with Ministry of Local Government, Rural Development and Co-operatives (MoLGRDC) responsible for provision of primary health care [13], while the Ministry of Health and Family Welfare (MoHFW) is responsible for providing secondary and tertiary health care [14]. The MoLGRDC currently contracts out the primary healthcare provision to Non-Government Organizations (NGOs) through the Urban Primary Health Care Service Delivery Project (UPHCSDP) [15]. In previous studies researchers demonstrated that MoLGRDC lacked capacity to provide health services effectively as there were weaknesses in governance and regulations, budget, referral linkages with secondary and tertiary healthcare facilities, and there were problems related to structure and coordination [16, 17]. However, not much is known about the maternal, infant and young child nutrition (MIYCN) services provided through the public primary health facilities in urban areas. The aim of the current study is to explore the facilitators and challenges related to nutrition service delivery in the public sector in urban Bangladesh from both user and provider perspectives.

## Methods

### Study design and site

This study was part of a broader cross sectional, mixed methods formative research designed to understand the urban nutrition service delivery at facility and community-levels. For our current paper we focused on qualitative data from public sector primary care facilities and their catchment area. The community-level data were collected from the catchment areas of two purposively selected UPHCSDP clinics in Dhaka North and South city corporations. We observed nutrition service provision in a convenience sample of 8 UPHCSDP clinics. The study was carried out from April to July 2019.

### Context

In the urban areas, primary healthcare is provided through three tiers: (1) Comprehensive Reproductive Health Care Center (CRHCC), (2) Primary Health Care Centre (PHCC) and (3) outreach services. CRHCC includes ANC, postnatal care (PNC), delivery services, family planning, menstrual regulation, post abortion care, immunization and limited curative services. PHCC includes both static and satellite clinics where ANC, PNC, family planning, menstrual regulation, immunization and limited curative care are provided through static clinics, and immunization, detection and referral of complicated cases are provided through satellite clinics. In terms of outreach, the community health workers mainly register couples within reproductive age, identify and register pregnancy, provide information about the facilities and encourage mothers to visit healthcare centers. In all levels of primary health care, nutrition information and services are supposed to be provided.

### Data collection

The respondents for the qualitative assessment were selected purposively from both slum and non-slum areas within the catchment area of UPHCSDP clinics. We used a free listing exercise to identify the currently accessible, trusted and available communication channels that mothers use to seek MIYCN information and services. SSI were used to explore experience of using nutrition services in the UPHCSDP clinics from mothers’ perspective. To explore challenges in delivering MIYCN services, we conducted KII with program managers and health care providers of UPHCSDP clinics (Table 1). Data collection and analysis were performed in an iterative fashion. Data collection continued until saturation was reached. For SSI we Initially conducted 36 interviews but continued with seven more SSI to reach data saturation.

Initially, the health care workers helped us to identify respondents. They provided a list of pregnant women and mothers of young children. We selected some participants purposively from the list. In some cases, we used snow ball technique where a recruited mother or pregnant woman helped identify other mothers in the area based on our criteria [18]. The selection criteria included women’s life stage (pregnant or caregivers of 0 − 24 months-old-children), being residents of UPHCSDP clinic catchment area, and area type (slum or non-slum). None of the mothers approached for interviews refused to participate. The participants for the free listing exercise were selected purposively from SSI participants. Free listing and SSIs were conducted at respondents’ homes and KIIs with health care providers were carried out at health facilities. Participants of KII were selected purposively.

**Table 1:** Data collection tools and sample size

Methods	Respondents	Sample Size
Free listing	Pregnant women	5
	Mothers of children 0–24 months of age	10
Semi structured interview (SSI)	Pregnant women	12
	Mothers of children 0–6 months of age	12
	Mothers of children 6–24 months of age	19
Key informant interview (KII)	Program managers	2
	Health care providers	4
Service delivery observation	Pregnant women	40
	Mothers of children 0–24 months of age	35

The data were collected by a research team composed of sociologists and anthropologists, with at least five years of experience in collecting qualitative data. KII and SSI were conducted using separate guidelines. The SSI guideline for service recipients covered topics such as perception of availability of services, satisfaction with the service provision (cleanliness, behavior, waiting time), and reason for not using the service (if service was not used). The KII guideline for the service providers covered topics such the types of nutrition services provided by UPHCSDP; barriers in providing growth monitoring, counselling, IFA and calcium distribution; and provision of reminders and referrals. The guidelines for both KII and SSI were pre-tested in a different area (not part of the study) for finalization.

To assess the nutrition service delivery, we conducted structured observations during ANC, immunization and sick child visits. From each facility, nutrition service provision for 40 pregnant women (5 per primary facility) was observed on a first come first served basis. For mothers of young children 40 observations in total (5 per primary facility) was planned in the same manner as the pregnant women. However, we were able to observe 35 cases of service provision as in some cases service providers did not allow us to observe service provision. We used a structured checklist for observation of the service provision which was adapted from SPRING 2015 Tool [19]. The checklist for ANC observation included availability of dedicated space for counseling, anthropometric measurement, counseling on diet and breastfeeding. The checklist for children included growth monitoring (length/height and weight), and provision of nutrition counseling.

### Ethics statement

Local ethical clearance was obtained from the Ethical Review Committee of International Centre for Diarrhoeal Disease Research, Bangladesh (icddr,b) prior to the data collection (icddr,b protocol #: PR-18,092). The Institutional Review Board of Family Health International 360 acknowledged the study as exempt (Project #: 1245006-3). Researchers obtained informed written consent from each of the participant before data collection. The purpose of the study, anticipated duration of the interview, confidentiality and the right to withdraw at any phase of the study were clearly explained before each interview was conducted. As this study involved human participants, all of the procedures were followed in accordance with the relevant ethical guidelines and regulations, such as the Declaration of Helsinki.

### Data analysis

**Fig. 1:**
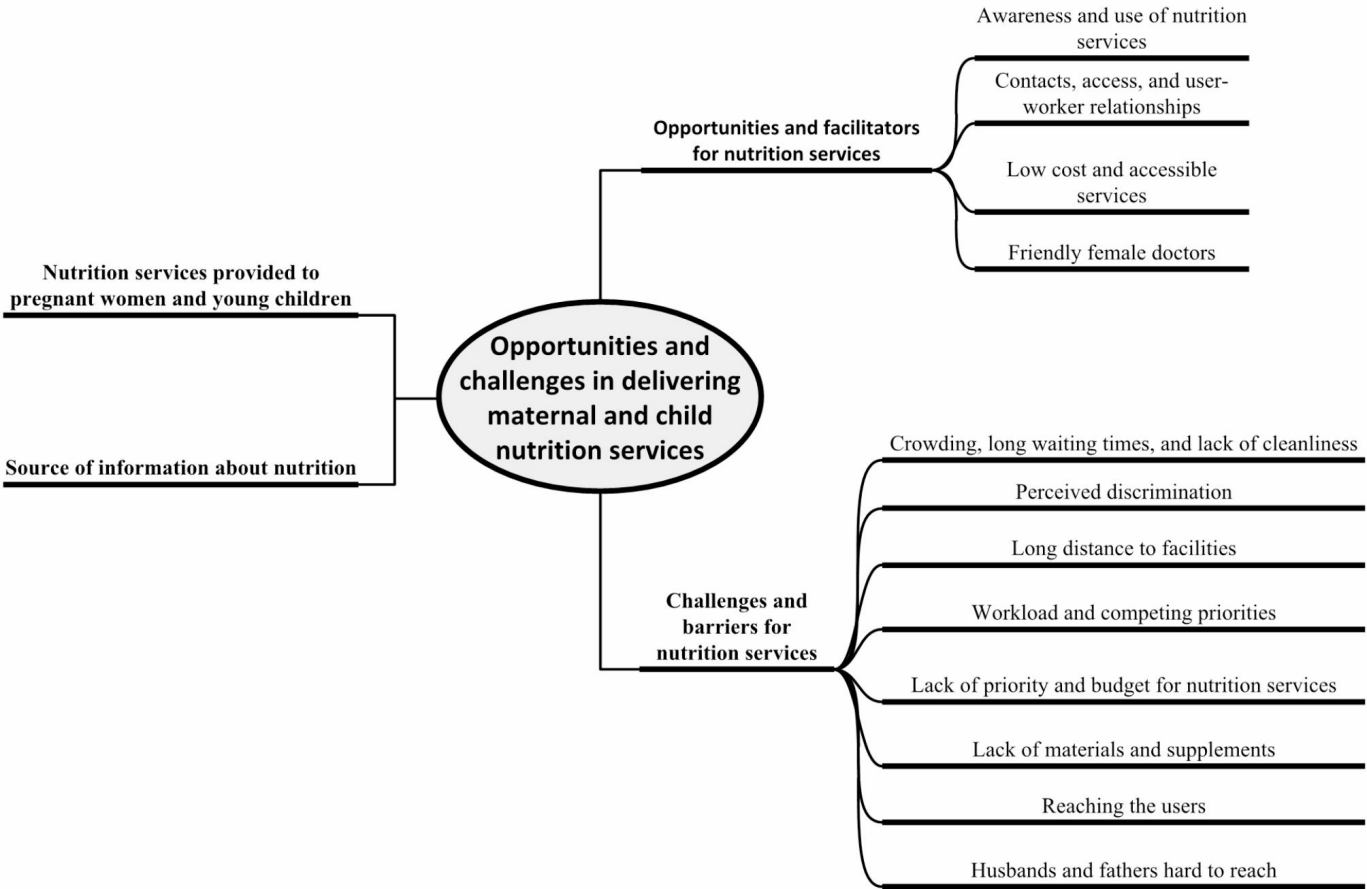
Themes of qualitative inquiry

All SSIs and KIIs were audio recorded and transcribed verbatim in Bengali. A thematic analysis was conducted [20]. We used repeated readings of the transcripts to gain an understanding of the data. Two researchers experienced in qualitative research coded the transcripts independently. The investigators crosschecked each other’s coded transcripts and met as a group to resolve any discrepancies in coding. Themes were identified, amended, refined and labelled (Fig. 1). Regular peer debriefing was conducted within the study team to help understand the issues and to interpret the findings. NVivo 10 was used for data management [21]. Data from free listing and observation were analysed in Microsoft Excel.

## Results

### Background characteristics of the participants

Most pregnant women and mothers were between 18 and 30 years of age, had some years of schooling, and were housewives. Many received nutrition services although most mothers did not know about nutrition services provided by the UPHCSDP clinics nearby. Most of the mothers and pregnant women had access to a mobile phone and television was the most used media while very few reported using newspapers or the internet. All service providers were older than 28 years, and had more than 6 years of education (Table 2).

**Table 2:** Characteristics of participants

Characteristics	Pregnant women (n = 12)		Mothers (n = 31)	Service providers (n = 06)
***Age group***				
18–22	6		13	0
23–27	5		10	0
28+	1		8	6
***Years of schooling***				
0–5	7		14	0
6–10	3		14	4
11+	2		3	2
***Parity***				
0	7		N/A	N/A
1–2	4		24	N/A
2+	1		7	N/A
***Profession***				
service	0		0	6
Housewives	12		31	N/A
***Residence***				N/A
Poor settlement	6		16	
Non-poor settlement	6		15	N/A
***Received Nutrition services***				N/A
Yes	7		10	
No	5		21	N/A
***Knowledge about nutrition services at UPHCSDP***				N/A
Yes	7		20	
No	5		11	N/A
***Mobile access (multiple count)***				N/A
Personal		6	23	
Family		12	27	N/A
No access		0	4	N/A
***TV***				N/A
Yes		6	25	
***Newspaper***				N/A
Yes		0	2	
***Internet***				
Yes		0	2	N/A

### Nutrition services provided to pregnant women and young children

All clinics had dedicated space for nutrition counseling and the health care providers greeted women with respect and dignity. There were gaps in counselling pregnant women about diets and breastfeeding. During the pediatric visits, 26 out of 35 children were weighed, but height/length was measured for only 6 children (Table 3).

**Table 3:** Nutrition services provided to pregnant women and children during observations in public primary healthcare clinics

Observation points	N (40)
***ANC visits***	
Dedicated space for nutrition counseling	40
Greeted clients with respect and dignity	40
Weight taken	36
Advised on diet	36
Advised on breastfeeding	6
**Pediatric observation**	**N = 35**
Weight measured	26
Weight checked against growth chart	14
Height measured	6
Used register book	29
Mothers/caregivers were advised on	
Frequency of feeding	27
Exclusive breastfeeding	14
Continued breastfeeding	18
Introduce solid/semi solid foods	16
Dietary diversity	17

### Sources of information about nutrition

In the free listing of the sources of nutrition information, mothers and pregnant women mentioned family members, relatives, community health workers (Family Welfare Assistants [FWAs], Health Assistants [HAs], service promoters), TV, internet, social media (Facebook), public health care provider and doctors as their sources of information about MIYCN. Only mothers from non-poor settlements mentioned internet and social media.

Despite recalling a variety of sources for nutrition information, not all sourceswere equally trusted. For pregnant women and mothers of young children, older females and husbands (family), and health workers and doctors (health sector) were the most trusted sources of information. Family members were trusted due to their role, easy access, belief that they wish the mother well, and good relationship. For healthcare providers, mothers believed that they were experts in the topic and therefore, can be trusted as a source of information. In terms of the media, TV was the most used and trusted source of information.

### Opportunities and facilitators for nutrition services

#### Awareness and use of nutrition services

Most mothers knew about UPHCSDP satellite clinics, but some of them didn’t know about the static health centers. Many mothers and a few pregnant women were unaware of the health and nutrition services provided by static primary healthcare clinics.

Among the participants, 7 pregnant women and 10 mothers reported utilizing the services of urban primary healthcare clinics (Table 2). The pregnant women visited the clinic for ANC checkups and the mothers mostly visited the clinic for vaccination and the treatment of sick children. Most of the respondents who used the clinics found the service useful, and were satisfied with the behavior of health care providers. As one pregnant woman said:

*“They said that my baby’s (fetus) weight was low. They advised me to take nutritious food and a lot of water daily. I am trying to follow their instructions. The providers were helpful and I found their advice useful.”* (Pregnant woman, slum, SSI-8).

#### Contacts, access, and user-worker relationships

The UPHCSDP clinic cadre who conducted household visits were the Service Promoters and FWAs. They were appointed to visit the designated catchment to identify the poor, list pregnant women and encourage mothers of young children to come for vaccination. These field staff also assisted at satellite clinics held at the community level to provide immunization and some family planning services. The frequent contact with the community allowed health workers to develop relationships and encourage people to avail of clinic services. As one clinic manager said,

*“Our health workers go on rounds to every ward (administrative unit) every day. They go house to house to provide health messages to pregnant and lactating mothers…they identify these mothers, fill up follow-up forms and encourage them to avail services. They (health workers) go often to encourage (mothers). They encourage the family members also. These constant contacts make the clinic attractive to people.”* (Project manager, KII-1).

#### Low cost and accessible services

The health care providers also spoke about the low cost of the services as an important reason for people’s preference for the UPHCSDP clinics. They thought that the poor were especially attracted to their clinic due to the low cost of services. As one health worker explained

*“Our clinic is low-cost. With only 40 takas they can get blood pressure and weight measurement, consultation with a gynae doctor(gynaecologist) and counselling… for each of the services they don’t have to pay more than 5 takas so why would a mother not come (to the clinic)? We also provide medicine at 10% discount. If you went to the private sector for these services, you would pay thousands of takas”* (FWA, KII-6).

Similar findings about low cost of services were also mentioned by users of UPHCSDP clinics.

#### Friendly female doctors

Health service providers highlighted that the friendly atmosphere of the clinic adapted to target women made the services attractive. The clinic employed female doctors to ensure that the female clients were comfortable discussing their reproductive health issues. As one clinic manager explained

*“Women use our services more. We have good doctors…having female doctors is an important aspect of our service. I think it creates a comfort zone for mothers…they can talk to the doctors easily”.* (Project manager, KII-3)

### Challenges and barriers for nutrition services

#### Crowding, long waiting times, and lack of cleanliness

Some pregnant women from non-poor settlements cited crowding, long waiting time and unclean spaces as reasons for not visiting the UPHCSDP clinics. They mentioned that private facilities met their expectations related to service provider’s behavior and facility cleanliness better. According to a pregnant woman:

*“It (urban primary health care clinic) was crowded and untidy. There was no queue in front of the physician’s rooms and no discipline. We have to wait a long time. Thus, I did not visit the urban health centre for the second time.”* (Pregnant woman, non-slum, SSI-31).

#### Perceived discrimination

A few women from poor settlements (red card holders) spoke about facing discrimination while receiving treatment from the clinics. The clinics provided “RED” cards for the ultra-poor that allowed them to receive free treatment. However, card holders claimed that they had to wait for a longer period than the non-card holders as the service providers prioritized paying patients.

No, I don’t have to pay (consultation fees), I have a red card. Most people have to pay there (at the health facility). Those who pay get served early. Those who go for free get less priority…we have to sit and wait for a long time. (Pregnant woman, slum, SSI-7)

#### Long distance to facilities

Two mothers, who did not visit the urban primary healthcare clinics for services despite knowing about them, pointed to the distance of the clinic from home as a barrier. When women have to incur travel costs to use the clinic, they often preferred visiting facilities that were nearby.

*“Actually, distance is a problem. I know that there is a clinic (urban primary healthcare clinic) but I have never visited there as the clinic is far away from our house. If we want to go there, we have to pay a lot. In our area, there is a clinic of BRAC, where pregnant mothers and children get services. Often women in our area visit nearby private facilities.” (Mother, slum, SS1-1)*.

#### Workload and competing priorities

The health care providers who provided services through the different clinics mentioned that they were overburdened with multiple responsibilities and were not able to spend a lot of time with the pregnant women and mothers of young children. They used their time to provide ANC, PNC, delivery, immunization and curative care but nutrition services were not prioritized during the visits. Although FWAs conducted household visits to promote use of contraception, and encourage families to bring children for immunization, providing nutrition services especially nutrition counselling during home visits was not their priority. When we asked if it was possible for them to provide nutrition education, health workers thought it would be difficult to provide good quality nutrition counselling in the current situation. As one project manager said:

*“If I could show them (mothers) how to cook and feed…talk to them about why they should feed in a way that it gets registered into their head, it would be best. We do tell them but what we do is not enough. We have one doctor who has to do so much. How can she also provide nutrition counselling?”* (Project manager, KII-1).

The workload and competing priority were explained by the program manager in the following way:

*“Nutrition is provided as a part of other services… we are not working on just nutrition… we are not focusing on it (nutrition). It is important to identify problems first and then if there was a pool of trained health workers or counsellors providing individualized service, it would have been good.”* (Project manager, KII-3).

A health worker recommended specifying a day for counselling so that good quality nutrition and health counselling could be provided

*“I think if only nutrition services are provided one day a week…if other services are not provided (on that day), then good quality nutrition services can be provided. It takes time (counselling). If many people come for different services, there is less time for counselling and other nutrition services.”* (FWA, KII 2).

#### Lack of priority and budget for nutrition services

Nutrition service provision also suffered from lack of priority by both the donors of UPHCSP and implementing NGOs. The lack of priority for nutrition services was attributed to the bidding process that the NGOs went through. To be a provider of urban primary health care, there was a bidding process through which vendors offering the lowest costs received the contract. To be competitive, NGOs often cut corners on personnel and training and as a result, when they received the contract, they had very little flexibility to focus on what was perceived to be “less important” activities based on their reporting requirements and the interest of the donor. According to a manager:

*“Now we do not have any training (on nutrition) in our budget. We did not keep the cost of the training (in the project budget) …We kept the cost low because we tried to win the bid. If our demonstrated cost was higher, we would not get it (contract)…so we didn’t keep money for nutrition training… it will increase the cost.”* (Project manager, KII-3).

The lack of priority and inadequate budget resulted in inadequate training (both pre-service and refreshers) of the service providers and a weak monitoring system. The program manager spoke about the importance of capacity development of health workers through pre-service and refreshers training, supportive supervision to ensure the delivery of good quality nutrition services.

As the program manager of a PHCC said

*“Nutrition can be added (to primary care) but it is important to provide good quality training. However, only training is not sufficient, they (health workers) have to be monitored and their knowledge has to be constantly upgraded.”* (Project manager, KII-1).

#### Lack of materials and supplements

Some health workers talked about the inadequate supply of micronutrient supplements and behavior change communication (BCC) materials in the health facility being a barrier to providing good quality nutrition services. The health workers reported using BCC materials such as food models and handouts to educate people about animal-source foods, vitamin A and iron-rich foods. The project manager described the shortage of BCC materials in the following way:

*“Every clinic has demonstration materials… we had models (of foods) to show mothers. We also had flipcharts and leaflets. We can only use what we have in the facility …when we run out we cannot provide demonstrations. Often these materials are not available to us.”* (Project manager, KII-1).

Health workers from some facilities mentioned sporadically running out of nutrition supplements such as iron folic acid while those in other facilities said that they had adequate supplies. Health workers felt that being able to provide some materials and supplements to the mothers helped mothers to accept their counselling. They felt that it was important to be able to give the clients something to highlight the nutrition services.

#### Reaching the users

During the outreach, health workers talked about not being able to reach urban mothers or key family members as a barrier to providing nutrition services. The lack of access to the clients meant that continuity of care was disrupted and sometimes support for the mothers from their families could not be ensured. One of the biggest problems faced by health workers was the constant migration of people. As the project manager explained:

*“Whether they are poor or middle-income, the families in Dhaka are mostly floating (migrating from one place to the other). If we counsel one person today, we don’t know that they will stay in our area tomorrow…you can say that everyone has a mobile phone and they can receive a message…I do not think we can help them adequately if we cannot reach them.”* (Project manager, KII-1).

In middle income settlements, especially among people who live in flats, concerns about security reduced access to clients. This lack of access created problems with identifying potential clients and providing them with services and reminders. As a health worker said -

*“Sometimes I reach a household and they do not even open the door or close the door after seeing me. The caretakers (of a building) refuse to let us in saying that they will lose their job if anything happens…of course, we face these problems.”* (FWA, KII-6).

#### Husbands and fathers hard to reach

Reaching out to family decision-makers, such as husbands, to ensure that mothers were supported regarding nutrition was also a challenge in the urban areas as many decision-makers work long hours and were only available during the evening.

*“Husbands are hard to reach…we get their numbers for counselling and ask them for their free time so that we can call… it’s hard as they work and don’t want to spare us time.”* (FWA, KII-2).

This finding was explained further in detail by the project manager

*“We can’t reach the family effectively as the fathers can’t be reached… Some are vegetable sellers, rickshaw pullers, garment workers or drivers…if we could organize a meeting at night with the help of male health workers that would be really helpful”.* (Project manager, KII-3)

Health workers mentioned that having flexible work hours and male health workers (for security) would help them to work in the evenings.

The comparison of favorable factors and barriers, as reported by the users of nutrition services and providers, revealed agreements, contrasts, and unique perspectives. A key favorable factor was the agreement between providers and users of services included clients’ satisfaction related to usefulness of nutrition advice, and helpfulness of providers. A key barrier contrast between providers and users relates to a complex dynamic involving the distance to the clinic and related cost of service. Providers perceive advantage of low-cost public services that would translate into preference by users over high cost private services. In reality and as reported by a study participant, there are instances of women preferring to visit private facilities that are located near their homes and not low-cost public facilities located far away. Our analysis revealed a contrast between levels of providers, managers and community health workers. A manager reported that daily household visits by community health workers translate into trust relationships that include access to family members but the community health workers reported instances of distrust with specific household residents not opening the door or building caretakers not letting them in. Providers agreed that access to husbands and fathers is a challenge as they were not available at the time that household visits occur and often did not respond to phone calls. Unique barrier from user perspective that did not emerge from service providers included dissatisfaction with services due to crowding, long waiting times, unclean facilities, perceived discrimination.

## Discussion

In this study, we explored the challenges and opportunities of delivering nutrition services through urban primary health care facilities in public sector from both service provider and recipient perspectives. In a recently published paper researchers reported that there were gaps in the nutrition service delivery and that client satisfaction with public sector services was lower than that of private and NGO sector health facilities for MICYN services [22]. However, this study did not delve into the barriers and facilitators of service provision in the public sector. The current qualitative study was designed to fill the gap in knowledge about nutrition service delivery through public sector in urban areas. The findings from our study will be instrumental in understanding the challenges of nutrition service delivery in the urban public sector and thinking about how to address these challenges.

We found that there were gaps in anthropometric measurement for infants and young children. Although some children were weighed and their height was measured, in very few cases the anthropometric measurement was compared with growth chart; which meant that the anthropometric measurement was not adequately used to have a conversation about growth and feeding of the children. Similar findings about inadequate anthropometric measurement have been reported from both rural and urban areas of Bangladesh [23]. This is a missed opportunity as growth monitoring and promotion is intended to create an opportunity for interaction between healthcare workers and mothers regarding health and wellbeing of their children [12, 24]. There were also gaps in the counselling for pregnant mothers related to diets during pregnancy and breastfeeding indicating that mothers were not receiving adequate nutrition information from primary health services. Studies have shown that good quality nutrition counselling was effective in changing infant and child feeding practices [25–27]. It is therefore, important to invest in developing skills of health workers and creating enabling conditions for counselling in the public primary care facilities. In this regard, service providers mentioned that it is hard to provide adequate nutrition counselling while providing other curative services and the possibility of creating a space for well child visits in their facilities. In a recently published paper creating an opportunity for well child visits within primary health care setting was deemed feasible [28]. However, it is important that the well child visits are implemented with adequate training of the workforce and consideration of how this will impact other services.

To improve nutrition service utilization through urban primary care facilities in the public sector, it is important that the challenges such as distance, waiting time, cleanliness and outreach to the community are addressed. In the previous study on a similar population, researchers also reported that client satisfaction with public facilities was lower than with other health facilities and that long waiting times and lack of cleanliness were important parts of client satisfaction [22]. Researchers reported high caseloads in public facilities leading to long waiting time and consequently, less satisfaction with the services received [29, 30]. These findings indicate that it is critical to organize and design service delivery with the population expectation and dynamics in mind. In urban areas, it is important to consider the service hours, devise alternate ways of reaching critical community members and design the services for frequently migrating populations so that continuity of care can be maintained.

To strengthen nutrition service delivery through public urban primary care facilities, one of the most significant issue was the lack of prioritization of nutrition services during the design of UPHCSDP project. The lack of attention to nutrition service provision started from the contracting and bidding process where the vendors with the lowest budget was awarded the contract. This lead to the vendors cutting corners for nutrition service delivery which in turn lead to inadequate resources for delivery of quality nutrition services [17]. Despite mainstreaming nutrition into health services, the lack of priority given to nutrition during implementation have been reported from Bangladesh and other low and middle-income countries [31, 32]. In the urban Bangladesh, primary health care provided by public sector is run as a project with external funding and suffered from the lack of coordination between MoLGRDC and MoHFW [16]. The challenges of urban PHC also have implications on nutrition service delivery in urban areas. In previous studies researchers have shown that nutrition has received political attention and commitment and both high-level and sector specific policies exist to support nutrition programming [33]. However, in the urban areas nutrition service delivery needs to be prioritized within the design of health service delivery and adequate resources should be allocated to strengthen the quality of nutrition services.

Urban primary healthcare is designed to support the needs of 52 million urban population of Bangladesh [1]. The PHC service provided by the public sector is an important source of health services for the growing urban poor [34]. The inequity in health indicators and lack of utilization of health services by the urban poor indicates [8] that the public sector PHC service provision needs to improve significantly with special attention to the dynamics of urban population and their specific needs. In addition to the improvement of PHC, special attention needs to be paid to nutrition service delivery, which could increase client satisfaction and help increase health service delivery as well. The strengthening of PHC has the potential to contribute to reducing the impact of shocks to the health systems such as the COVID-19 pandemic which severely affected both service provision and utilization [35]. Addressing the nutrition needs of urban residents through strengthening the PHC will contribute to several Sustainable Development Goals (SDGs) including SDG 2, Zero hunger; and 3, Good health and well-being [36].

### Policy and programmatic implications

Based on our findings, we identified three areas where there were barriers that need to be resolved at the levels of service quality, community outreach and policy. We have summarized the barriers, favourable factors and opportunities of intervention in all three levels in the table below (Table 4).

**Table 4:** Policy and program implications of the findings

Areas of interest	Constraints and barriers	Favorable factors	Opportunities for intervention
Quality of services	- Long waiting hours - Lack of cleanliness - Lack of BCC materials -Inadequate training -Stock outs	- Providers are aware of the need of providing quality service -Trust in health care providers -Availability of female providers	- Engaging volunteers to provide health education at facility level -Utilize waiting time to provide health education and information (such as short films) -Develop a mechanism for monitoring health system readiness -Develop a mechanism for client feedback - Invest in providing regular pre-service training and supportive supervision to frontline health workers on nutrition -Design mechanisms (awards, incentives) to ensure that health workers provide good quality nutrition services -Create opportunity for well child visits
Awareness in the community	- Mothers were unaware of the services at the facility -Lack of continuity of care -Lack of access to clients -Inflexible design of program -Lack of resources	-Demand for the Nutrition services -Trust in the services provided - Providers are interested in reaching out to the clients	- Communication strategies need to be designed with the characteristics of urban population in mind - Mechanisms need to be designed to allow mothers to avail continuity of care - Create demand for well child visits
Policy and program	-Contracting process of NGOs do not have sufficient safeguards to ensure quality nutrition service delivery - Low program cost is prized over quality of service	-There is policy emphasis on nutrition - Nutrition has been integrated in the existing MNCH programs -Some nutrition indicators are incorporated in the monitoring reports	-Model for quality nutrition service delivery in urban areas should be developed -Contracting process for NGOs should be based on quality of nutrition service provision -Programs should be provided so flexibility to respond to the needs of the clients -Programs should be adequately budgeted to allow good quality nutrition services

### Strengths and weaknesses

The strength of our study was that we were able to collect data from multiple perspectives (mothers, caregivers and health care providers) which allowed us a holistic understanding of the barriers and opportunities for nutrition service delivery in urban areas. However, there were a few weaknesses of the research. From the supply side we only interviewed program managers and health care providers of UPHCPD facilities who did not have a lot of insights about the politics of PHC in the urban public sector which would have enriched our understanding of the context. Further we interviewed the health service providers in the clinic which might have biased their response to some extent.

## Conclusions

Our study demonstrated that there were important gaps in the readiness of primary care facilities in terms of equipment, capacity of healthcare providers and quality of service provided. From the community perspective, although there were clear needs for nutrition information and support for pregnant women and mothers of young children, few in the community knew about the nutrition services provided by the public sector facilities. Mothers reported challenges related to distance, waiting time and cleanliness that discouraged them from using public facilities in general. Finally, we found that nutrition needs to be prioritized during the design of urban primary healthcare so that adequate resources are located for maximizing coverage of quality nutrition services. As the urban population grows, it is important that nutrition services provided through urban primary health care in the public sector are prioritized so that Bangladesh can meet Sustainable Development Goal related to reduction of malnutrition by addressing the needs of the urban poor.

### Electronic supplementary material

Below is the link to the electronic supplementary material.

Supplementary Material 1

## Data Availability

Data are available upon reasonable request for researchers from Armana Ahmed (armana@icddrb.org), Head of Research Administration of icddr,b, and as per the data access policy of icddr,b.

## References

[1] Bangladesh Bureau of Statistics (BBS): Population and housing census 2022 Preliminary Report, Dhaka, Bangladesh BBS. ; 2022. Available from: https://sid.gov.bd/sites/default/files/files/sid.portal.gov.bd/publications/01ad1ffe_cfef_4811_af97_594b6c64d7c3/PHC_Preliminary_Report_(English)_August_2022.pdf. Accessed 17 Nov 2022.

[2] Bangladesh Bureau of Statistics(BBS): Changing Patterns of Urbanization in Bangladesh:An analysis of census data. Dhaka, Bangladesh: BBS. ; 2015. Available from: http://203.112.218.65:8008/WebTestApplication/userfiles/Image/PopMonographs/Volume-12_UM.pdf. Accessed 17 Jan 2022.

[3] Razzaque A, Iqbal M, Hanifi S, Mahmood SS, Mia MN, Chowdhury R, Mustafa A, Bhuiya A, Majumder M, Hakim A (2019). Slum health in Bangladesh: insights from health and demographic surveillance.

[4] Razzaque A, Clair K, Chin B, Islam MZ, Mia MN, Chowdhury R, Mustafa AG, Kuhn R (2020). Association of time since migration from rural to urban slums and maternal and child outcomes: Dhaka (north and south) and Gazipur City corporations. J Urban Health.

[5] Assaf S, Juan C (2020). Stunting and Anemia in children from Urban Poor environments in 28 low and middle-income countries: a Meta-analysis of demographic and Health Survey Data. Nutrients.

[6] Mberu BU, Haregu TN, Kyobutungi C, Ezeh AC (2016). Health and health-related indicators in slum, rural, and urban communities: a comparative analysis. Glob Health Action.

[7] Bangladesh Bureau of Statistics (BBS) and United Nations Children’s Fund (UNICEF): child and youth wellbeing survey in Bangladesh. Dhaka, Bangladesh: BBS and UNICEF. ; 2017. Available from: https://bbs.portal.gov.bd/sites/default/files/files/bbs.portal.gov.bd/page/b343a8b4_956b_45ca_872f_4cf9b2f1a6e0/Report%20on%20CWS%202016%20Urban%20areas%20in%20%20%20Bangladesh.pdf. Accessed 18 Jun 2022.

[8] National Institute of Population Research and Training (NIPORT): Urban Health Survey 2021. Dhaka, Bangladesh:NIPORT. :2022. Available from: https://www.niport.gov.bd/sites/default/files/files/niport.portal.gov.bd/publications/66c4accd_4c6a_4aab_902c_781a77aa8768/2023-01-30-06-04-9310fc1f3902cdd03884124c600ddc8d.pdf. Accessed 19 Jun 2022.

[9] Pelletier D, Shekar M, Du L, Kostermans K: The Bangladesh Integrated Nutrition Project Effectiveness and Lessons. Bangladesh Development Series,paper no. 8., Washington DC. World Bank Group; 2005. Available from : http://documents.worldbank.org/curated/en/592641468013784606/The-Bangladesh-Integrated-Nutrition-Project-effectiveness-and-lessons. Accessed 27 Jan 2022.

[10] Hossain SM, Duffield A, Taylor A (2005). An evaluation of the impact of a US $60 million nutrition programme in Bangladesh. Health Policy Plan.

[11] Saha KK, Billah M, Menon P, El Arifeen S, Mbuya NV (2015). Bangladesh National Nutrition Services: assessment of implementation status.

[12] Billah SM, Saha KK, Khan ANS, Chowdhury AH, Garnett SP, Arifeen SE, Menon P (2017). Quality of nutrition services in primary health care facilities: implications for integrating nutrition into the health system in Bangladesh. PLoS ONE.

[13] Government of The People’s Republic of Bangladesh (1983). The Dhaka City Corporation Ordinance, 1983.

[14] Government of The People’s Republic of Bangladesh. : Rules of Business.Cabinet Division ed., vol. Schedule I: Allocation of Business Among the Different Ministries and Divisions. Cabinet Division;2014. Available from: https://cabinet.portal.gov.bd/sites/default/files/files/cabinet.portal.gov.bd/legislative_information/13237291_40e2_4538_84ab_37ec65fe11ea/Allocation%20of%20Business%20R-20140001.pdf. Accessed 27 Jan 2022.

[15] Ahmad A. Provision of primary healthcare services in urban areas of Bangladesh: the case of urban primary healthcare project. Working paper. Lund, Sweden: Lund University;2007. Available from: http://hdl.handle.net/10419/259941. Accessed 27 Jan 2022.

[16] Ramesh G, Dhushyanth R, Secci F, Sadia C, Frere J (2018). Health and nutrition in urban Bangladesh: social determinants and health sector governance.

[17] Islam R, Hossain S, Bashar F, Khan SM, Sikder AA, Yusuf SS, Adams AM (2018). Contracting-out urban primary health care in Bangladesh: a qualitative exploration of implementation processes and experience. Int J Equity Health.

[18] Patton MQ (2015). Qualitative evaluation and research methods.

[19] SPRING: Tool for Rapid Evaluation of Facility-Level Nutrition Assessment, Counseling, and Support: A User’s Guide. SPRING. ;2015 Available from: https://www.spring-nutrition.org/publications/tools/tool-rapid-evaluation-facility-level-nutrition-assessment-counseling-and-support. Accessed 29 Jan 2022.

[20] Braun V, Clarke V (2006). Using thematic analysis in psychology. Qual Res Psychol.

[21] Castleberry A. NVivo 10 [software program]. Version 10. QSR International; 2012. Am J Pharm Educ. 2014;78(1).

[22] Islam Anne F, Akter SM, Sheikh SP, Ireen S, Escobar-DeMarco J, Kappos K, Ash D, Rasheed S (2022). Quality of nutrition services in primary health care facilities of Dhaka city: state of nutrition mainstreaming in urban Bangladesh. PLoS ONE.

[23] Chowdhury MRK, Khan HT, Rashid M, Mondal MNI, Bornee FA, Billah B (2022). Prevalence and correlates of severe under-5 child anthropometric failure measured by the composite index of severe anthropometric failure in Bangladesh. Front Pediatr.

[24] Government of The People’s Republic of Bangladesh: Community-based health care operational plan (2011 – 2016). Dhaka, Bangladesh; Ministry of Health and Family Welfare. ; 2011. Available from: https://maternalnutritionsouthasia.com/wp-content/uploads/National-Operational-Plan-Community-Based-Health-Care.pdf. Accessed 30 Jan 2022.

[25] Rana R, McGrath M, Sharma E, Gupta P, Kerac M (2021). Effectiveness of breastfeeding support packages in low-and Middle-Income Countries for Infants under six months: a systematic review. Nutrients.

[26] Kim SS, Nguyen PH, Tran LM, Alayon S, Menon P, Frongillo EA (2020). Different combinations of behavior change interventions and frequencies of interpersonal contacts are associated with infant and young child feeding practices in Bangladesh, Ethiopia, and Vietnam. Curr Dev Nutr.

[27] Mistry SK, Hossain M, Arora A (2019). Maternal nutrition counselling is associated with reduced stunting prevalence and improved feeding practices in early childhood: a post-program comparison study. Nutr J.

[28] Nguyen PH, Pramanik P, Billah SM, Avula R, Ferdous T, Sarker BK, Rahman M, Ireen S, Mahmud Z, Menon P. Using scenario-based assessments to examine the feasibility of integrating preventive nutrition services through the primary health care system in Bangladesh. Matern Child Nutr. 2022;e13366. 10.1111/mcn.13366. 10.1111/mcn.13366 PMC9218316 35508919

[29] Adhikary G, Shawon MSR, Ali MW, Shamsuzzaman M, Ahmed S, Shackelford KA, Woldeab A, Alam N, Lim SS, Levine A (2018). Factors influencing patients’ satisfaction at different levels of health facilities in Bangladesh: results from patient exit interviews. PLoS ONE.

[30] Andaleeb SS, Siddiqui N, Khandakar S (2007). Patient satisfaction with health services in Bangladesh. Health Policy Plan.

[31] Girard AW, Dzingina C, Akogun O, Mason JB, McFarland DA (2012). Public health interventions, barriers, and opportunities for improving maternal nutrition in Northeast Nigeria. Food Nutr Bull.

[32] Pelletier DL, Frongillo EA, Gervais S, Hoey L, Menon P, Ngo T, Stoltzfus RJ, Ahmed AS, Ahmed T (2012). Nutrition agenda setting, policy formulation and implementation: lessons from the Mainstreaming Nutrition Initiative. Health Policy Plan.

[33] Rasheed S, Roy SK, Das S, Chowdhury SN, Iqbal M, Akter SM, Jahan K, Uddin S, Thow AM (2017). Policy content and stakeholder network analysis for infant and young child feeding in Bangladesh. BMC Public Health.

[34] Albis MLF, Bhadra SK, Chin B (2019). Impact evaluation of contracting primary health care services in urban Bangladesh. BMC Health Serv Res.

[35] Nguyen PH, Sununtnasuk C, Pant A, Tran Mai L, Kachwaha S, Ash D, Ali M, Ireen S, Kappos K, Escobar-Alegria J (2021). Provision and utilisation of health and nutrition services during COVID‐19 pandemic in urban Bangladesh. Matern Child Nutr.

[36] United Nations. Sustainable Development Goals. New York, USA: UN Department of Economic and Social Affairs 2015. Available from: https://sdgs.un.org/goals. Accessed 13 March 2022.

