# Supplementary material for: Opportunities and challenges in delivering maternal and child nutrition services through public primary health care facilities in urban Bangladesh: a qualitative inquiry
Source: BMC Health Serv Res. 2023 Oct 27;23:1172. doi: 10.1186/s12913-023-10094-6 (PMC10612189; doi:10.1186/s12913-023-10094-6)
Supplement: Supplementary file 1 — Supplementary Material 1 [file 12913_2023_10094_MOESM1_ESM.pdf]

Guideline for SSI

Name:
Age:
Sex:
Education:
Profession:
Do you own a mobile phone?
How do you use it?
Do you have TV?
Do you have access to the internet?
Do you read newspaper?

- Which heath facility do you prefer to go to for health care during pregnancy? Why do you prefer this health facility?
- Have you used clinics of Urban Primary Health Care Service Delivery Project (UPHCSDP) for care seeking during pregnancy? Please explain the reason for not using the service (if service was not used)
- Do they provide any nutritional services? What are your experiences in receiving such services? [perception of availability of services, satisfaction with the service provision (cleanliness, behaviour, waiting time)]
- What should the nutrition services look like in these healthcare facilities? (probe: time taken, fees, referral, outreach)
- How would you like the health facility to reach you with information and reminders? (probe: use of media, how to develop trust in the info received)
- What would make you trust the information you receive from different sources? (probe media, institution, person)

KII Guideline for service provider/program personnel

Name:
Age:
Sex:
Education:
Job title:

- Please tell us the types of nutrition services you provide for the pregnant mothers and young children currently? Give us some examples (probe: growth monitoring, counselling, provision of IFA, Calcium or other products, reminder, referral)

- Do you face any barriers in providing such services? (probe: growth monitoring, counselling, provision of IFA, Calcium or other products, equipment, human resources, workload, patient flow, reminder, referral or others)
- Is there any challenge for pregnant women and young mothers in accessing nutrition services at your facility?
- What can help to improve the nutrition services you provide at present (probe: training, supplies, time, mandate)
- What do you think is a good way of reaching mothers with nutrition information and services (Probe: media, outreach, use of mobile phone, linking with community groups, community leaders)?
- Please suggest who can be engaged to strengthen the nutrition services provision through health care facilities (community group, community leader, others)?

Checklist for Facility Observation during ANC

1	Observation Starting time:	_________ (HH:MM(24 hours)
2	Observation End time:	_________ (HH:MM(24 hours)
**3**	**Information of Pregnant Women/ Mother of children**	
3.1	No of ANC Visit :	☐ 1st Visit------------------1<br>☐ 2nd Visit-----------------2<br>☐ 3rd Visit-----------------3<br>☐ 4 th Visit------------------4
3.2	Age of pregnant mother (in years):	____Years

4	**Information During Observation**	
Note: *Carefully observe the* ***ANC check-up session*** *conducted by the provider and put a (√) in the applicable box.*		
No	**Observation Points**	**Responses**
4.1	Does the facility have space dedicated for nutrition counselling?	☐ Yes ☐ No ☐ N/A
4.2	Does the health care provider **greet** the woman with respect and dignity?	☐ Yes ☐ No ☐ N/A
4.3	Is the pregnant women given any nutrition advice during ANC?	☐ Yes ☐ No ☐ N/A
4.4	Does the health care provider, or another staff, weigh and record the **WEIGHT** of the woman today?	☐ Yes ☐ No ☐ N/A
4.6	Does the health care provider, or another staff, examined **ANAEMIA** in eyes (Lower palpebral fissure) of the woman?	☐ Yes ☐ No ☐ N/A

5	What **ADVICE (S)** does the health care provider provide to the women today? *Put a (√) in the applicable box*	

5.1	To take more food	☐ Yes ☐ No ☐ N/A
5.2	To take balanced and diverse diet	☐ Yes ☐ No ☐ N/A
5.3	To take animal source foods	
5.4	To take seasonal/available fruits	☐ Yes ☐ No ☐ N/A
5.5	To take green/coloured vegetables	☐ Yes ☐ No ☐ N/A
5.6	To drink more water	☐ Yes ☐ No ☐ N/A
5.7	To take iodized salt	☐ Yes ☐ No ☐ N/A
5.8	Told to visit for **regular antenatal check-ups (ANC)**	☐ Yes ☐ No ☐ N/A
5.9	Told to take routine **iron and folic acid(IFA)**	☐ Yes ☐ No ☐ N/A
5.10	Told about the importance of **breastfeeding**	☐ Yes ☐ No ☐ N/A
5.11	Initiation of breastfeeding within one hour	☐ Yes ☐ No ☐ N/A
5.12	Immunization	☐ Yes ☐ No ☐ N/A
5.13	Provided Iron-Folate tablet (IFA)	☐ Yes ☐ No ☐ N/A
5.14	Provided Calcium tablet	☐ Yes ☐ No ☐ N/A
5.15	Does the health care provider explain when to return for a follow-up visit?	☐ Yes ☐ No ☐ N/A
5.16	Does the health care provider thank the women after the ANC session?	☐ Yes ☐ No ☐ N/A
5.17	Does the delivery packages include formula?	☐ Yes ☐ No ☐ N/A

*Please review the entire observation checklist for any **missing points**,*
Fill up with (√) in appropriate box and finish the observation

Observation Checklist for Sick Under-Two Paediatric services

1	Surveyor Name and ID: ____________________ ____/____/	5B02 Date: ____/____/______/

	General Information of health facility	Code
2	Observation Starting time:	________ (HH:MM(24 hours)
3	Observation End time:	________ (HH:MM(24 hours)
4	Facility code	________/______/
**5**	**Information on the under-two child**	
**5.1**	Age of child:	______|_____ in weeks
**5.2**	Sex of child:	o Male----------------1<br>o Female -------------2

Information on the under-two years child during *pediatric session*		
Note: Carefully observe the pediatric session conducted by the provider and put a (√) in the applicable box		
No	**Observation Points**	**Responses**
**6.1**	Does the health care provider have any IMCI register book?	☐ Yes ☐ No ☐ N/A
**6.**2	Does the health care provider, or another staff, weigh and record the **WEIGHT** of the child today?	☐ Yes ☐ No ☐ N/A
**6.3**	Does the health care provider, or another staff, measure height/ length and **record the height/length** of the child today?	☐ Yes ☐ No ☐ N/A
**6.4**	Does health care provider check child's weight against a **GROWTH CHART**?	☐ Yes ☐ No ☐ N/A
**6.5**	Does health care provider ask about **BREASTFEEDING**?	☐ Yes ☐ No ☐ N/A
**6.6**	Does health care provider ask whether the child takes any other **FOODS/FLUIDS?**	☐ Yes ☐ No ☐ N/A
**6.7**	Does health care provider ask about **FEEDING** during illness?	☐ Yes ☐ No ☐ N/A
**6.8**	Does the health care provider **provide Zinc** tablet? (for diarrhoea)	☐ Yes ☐ No ☐ N/A
**6.9**	Did the health care provider advise the caregiver on Exclusive breastfeeding??	☐ Yes ☐ No ☐ N/A
**6.10**	Does the health care provider explain the need to continue breastfeeding at home?	☐ Yes ☐ No ☐ N/A
**6.11**	Did the health care provider advise the caregiver on frequency of feeding?	☐ Yes ☐ No ☐ N/A
**6.12**	Does the health care provider use the IMCI chart booklet at any time during the management of the child?	☐ Yes ☐ No ☐ N/A

**6.13**	Does health care provider use any visual job aids for demonstrating IYCF practice?	☐ Yes ☐ No ☐ N/A
**6.14**	Use of BCC materials to raise awareness on nutrition	☐ Yes ☐ No ☐ N/A
**6.15**	Prescribe/Provide Vitamin-A capsules	☐ Yes ☐ No ☐ N/A
**6.16**	Did the health care provider advise the caregiver on Introduce solid/semi solid foods?	☐ Yes ☐ No ☐ N/A
**6.17**	Did the health care provider advise the caregiver on Dietary diversity?	☐ Yes ☐ No ☐ N/A

*Please review the entire observation checklist for any **missing points**,*
Fill up with (√) in appropriate box and finish the observation.

___________________________________***___________________________________
